# Matrine Induces Apoptosis in Human Acute Myeloid Leukemia Cells via the Mitochondrial Pathway and Akt Inactivation

**DOI:** 10.1371/journal.pone.0046853

**Published:** 2012-10-08

**Authors:** Shenghui Zhang, Yan Zhang, Yan Zhuang, Jiajie Wang, Jianqin Ye, Si Zhang, Jianbo Wu, Kang Yu, Yixiang Han

**Affiliations:** 1 Key Laboratory of Molecular Medicine, Ministry of Education, and Department of Biochemistry and Molecular Biology, Fudan University Shanghai Medical College, Shanghai, China; 2 Laboratory of Internal Medicine, The First Affiliated Hospital of Wenzhou Medical College, Wenzhou, China; 3 Department of Hematology, The First Affiliated Hospital of Wenzhou Medical College, Wenzhou, China; Emory University, United States of America

## Abstract

Acute myeloid leukemia (AML) is a hematological malignancy characterized by a rapid increase in the number of immature myeloid cells in bone marrow. Despite recent advances in the treatment, AML remains an incurable disease. Matrine, a major component extracted from Sophora flavescens Ait, has been demonstrated to exert anticancer effects on various cancer cell lines. However, the effects of matrine on AML remain largely unknown. Here we investigated its anticancer effects and underlying mechanisms on human AML cells *in vitro* and *in vivo*. The results showed that matrine inhibited cell viability and induced cell apoptosis in AML cell lines as well as primary AML cells from patients with AML in a dose- and time-dependent manner. Matrine induced apoptosis by collapsing the mitochondrial membrane potential, inducing cytochrome c release from mitochondria, reducing the ratio of Bcl-2/Bax, increasing activation of caspase-3, and decreasing the levels of p-Akt and p-ERK1/2. The apoptotic effects of matrine on AML cells were partially blocked by a caspase-3 inhibitor Z-DEVD-FMK and a PI3K/Akt activator IGF-1, respectively. Matrine potently inhibited *in vivo* tumor growth following subcutaneous inoculation of HL-60 cells in SCID mice. These findings indicate that matrine can inhibit cell proliferation and induce apoptosis of AML cells and may be a novel effective candidate as chemotherapeutic agent against AML.

## Introduction

Acute myeloid leukemia (AML) is a group of clonal hematopoietic stem cell disorders in which both failure to differentiate and overproliferation in the stem cell compartment lead to accumulation of non-functional cells termed myeloblasts [1]. The primary objective in treating patients with AML is to induce a complete remission and thereafter prevent relapse. Recently, though high-dose induction treatment plus allogeneic stem cell transplantation can acquire a high rate of complete remission, 5-year overall survival rate of patients with AML was about 30% [2], [3]. Incomplete eradication of leukemic stem cells which express the CD34 antigen and concomitantly lack lineage-associated markers (CD34^+^lin^−^) may ultimately contribute to relapse. Therefore, alternative treatments still need to be found for patients with AML.

Some natural products have been used as alternative treatments for cancers including AML because of their extensive biological activities and comparatively low toxicities [4], [5]. Matrine, an alkaloid extracted from Sophora flavescens Aif, is quinolizidine with four-loop and molecular formula of C_15_H_24_N_20_. Matrine has been found to exhibit many biological activities, such as anti-inflammation, anti-virus, anti-fibrosis, anti-arrhythmia, and immunosuppression, leading to wide clinical use in the treatment of viral hepatitis, liver fibrosis, heart arrhythmia and skin diseases in China [6]–[11]. Recently, intensive studies have shown that matrine possesses potent antitumor activities by inhibiting proliferation and inducing apoptosis of cells from gastric cancer, lung cancer, hepatocellular carcinoma, breast cancer, melanoma, leukemia, multiple myeloma [12]–[21]. In addition, matrine can also induce the differentiation of leukemia K562 cells [20], the migration of lung cancer A549 cells [15], or the invasion of breast cancer MDA-MB-231 cells [17]. Key mechanisms proposed for the antitumor effects of matrine include regulating the expression of proliferation- and apoptosis-related genes or proteins, such as eIF4E, E2F-1, Fas, FasL, Bcl-2, Bax, and caspases [12]–[21]. We have previously shown that matrine triggers apoptosis of human multiple myeloma cells via activation of the mitochondrial pathway [21]. Liu *et al* [19] demonstrated that matrine induced apoptosis in U937 cells via caspases activation and MAPK-independent pathways. However, the systematic scientific evaluation and its anticancer mechanisms on AML cell lines as well as primary AML cells remain elusive.

In the present study, we investigated the cytotoxic effects of matrine on AML cell lines HL-60, NB4, and U937 as well as primary AML cells obtained from patients with AML and its underlying mechanisms *in vitro* and *in vivo*. We found that matrine could induce apoptosis of AML cells by collapse of mitochondrial membrane potential (*Δψm*), release of cytochrome c (cyt c) from mitochondria to cytosol, reduction of the ratio of Bcl-2/Bax, activation of caspase-3, and down-regulation of p-Akt and p-ERK1/2.

**Figure 1:**
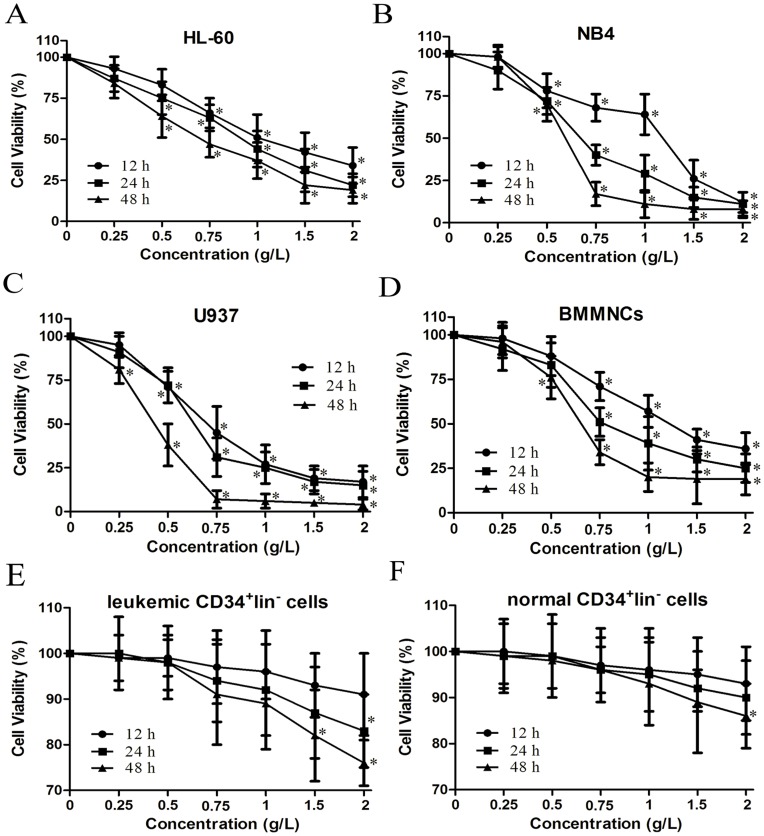
Effects of matrine on proliferation of AML cells, leukemic CD34^+^lin^−^ cells and normal CD34^+^lin^−^ cells. AML cell lines (HL-60, NB4, and U937), primary AML cells (BMMNCs), leukemic CD34^+^lin^−^ cells, and normal CD34^+^lin^−^ cells were treated with various concentrations of matrine for 12 h, 24 h and 48 h. Cell viability was assessed by MTT assay, and data were presented as the mean of at least three independent experiments. **A.** HL-60 cells. **B.** NB4 cells. **C.** U937 cells. **D.** BMMNCs. **E.** Leukemic CD34^+^lin^−^ cells. **F.** Normal CD34^+^lin^−^ cells.^ *^ *P*<0.05 vs the respective control.

## Materials and Methods

### Cell Lines and Primary Leukemic Cells

Human AML cell lines HL-60, U937 (obtained from the Cell bank of Chinese Academy of Sciences, Shanghai, China), and NB4 (gift from M.Lanotte [22]) were maintained in RPMI1640 medium supplemented with 10% fetal bovine serum (FBS; Gibco, Grand island, NY, USA). Primary human bone marrow mononuclear cells (BMMNCs) from 9 newly diagnosed and untreated patients with AML (M2, 2; M3, 2; M4, 2; M5, 3; the diagnosis and classification was established according to the French-America-British criteria) were isolated from BM aspirates by Ficoll-Hypaque centrifugation and subsequently cultured in RPMI1640 medium supplemented with 15% FBS. BMMNCs mainly contained primary AML cells and lymphocytes, in which primary AML cells could be distinguished from lymphocytes according to forward/side scatter. Leukemic CD34^+^lin^−^ cells were sorted from BMMNCs of patients with AML using the Diamond CD34 Isolation kit (Miltenyi Biotec, Bergisch Gladbach, Germany) according to the manufacturer’s protocols. Normal CD34^+^lin^−^ cells were sorted from BMMNCs of patients with non-Hodgkin’s lymphoma (NHL) without lymphoma involvement in BM. All protocols and experiments were approved by the First Affiliated Hospital of Wenzhou Medical College institutional review board for clinical experiments and use of human samples; written consents were obtained from all subjects participated in this study in accordance with the Declaration of Helsinki protocol.

**Figure 2:**
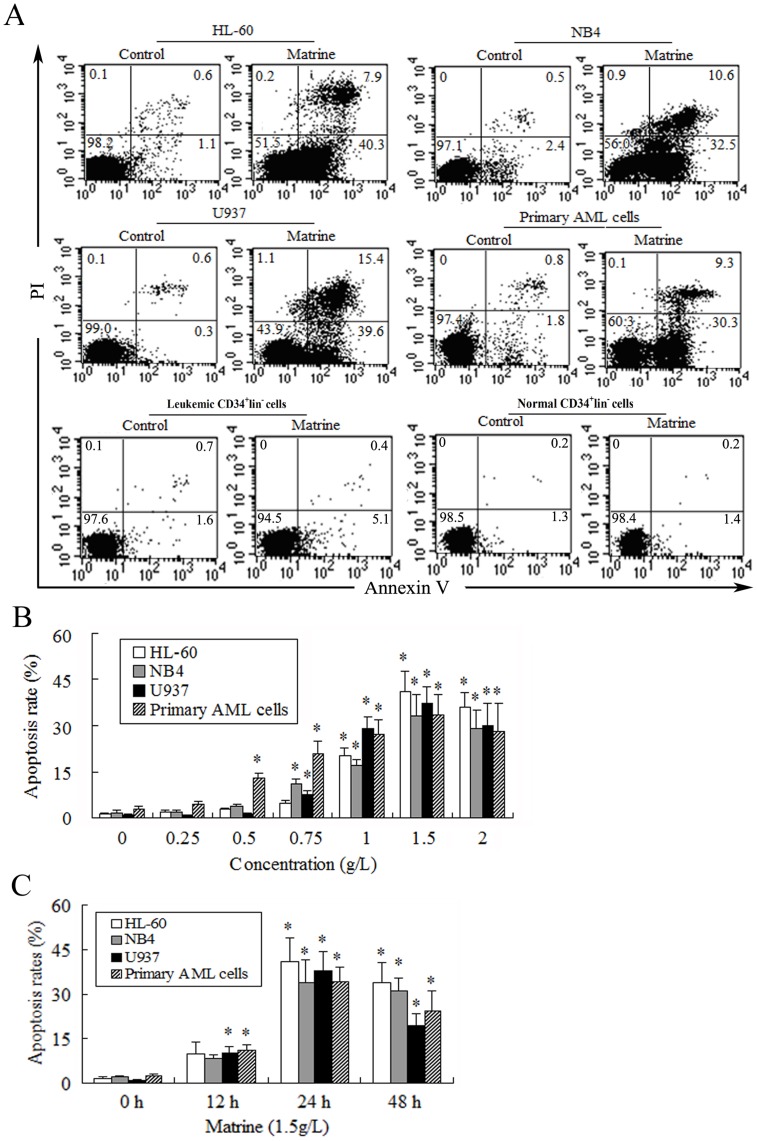
Matrine induced apoptosis in AML cells. **A.** AML cells, leukemic CD34^+^lin^−^ cells, and normal CD34^+^lin^−^ cells were treated with 1.5 g/L matrine for 24 h. The Annexin V-FITC binding and PI staining method was used to assess apoptosis, and representatives were shown. Both annexin V- and PI- negative (lower - left quadrant), annexin V- positive and PI- negative (lower - right quadrant), and both annexin V- and PI- positive (upper - right quadrant) cells were considered as the viable, early-phase apoptotic, late-phase apoptotic/necrotic cells, respectively. The percentage (%) of cells was described in each quadrant. **B.** AML cells were treated with various concentrations of matrine (0∼2 g/L) for 24 h. **C.** AML cells were treated by 1.5 g/L matrine for 12 h, 24 h and 48 h. The values represent the mean ± SD of at least three independent experiments. ^*^ *P*<0.05 vs the respective control.

### Cytotoxicity Assay

AML cells (HL-60, NB4, U937 and BMMNCs), leukemic CD34^+^lin^−^ cells, and normal CD34^+^lin^−^ cells were plated in 96-well microtiter plates and treated with various doses (0, 0.25, 0.5, 0.75, 1.0, 1.5, 2.0 g/L) of matrine (Tianyuan Biological Agent Plant, Xi’an, Shanxi, China) for 12 h, 24 h, and 48 h, and cell viabilities were assessed using the 3-(4,5-dlmethylthiazol-2-yl)-2,5-diphenyltetrazolium bromide (MTT) (Sigma-Aldrich, St Louis, MO, USA) assay. The absorbance (A) was read at 490 nm using an ELISA reader (ELx800; Bio-Tek Instruments, Winooski, VT, USA). Cell viability rate (%) = A_490, matrine_/A_490, control_×100%.

**Figure 3:**
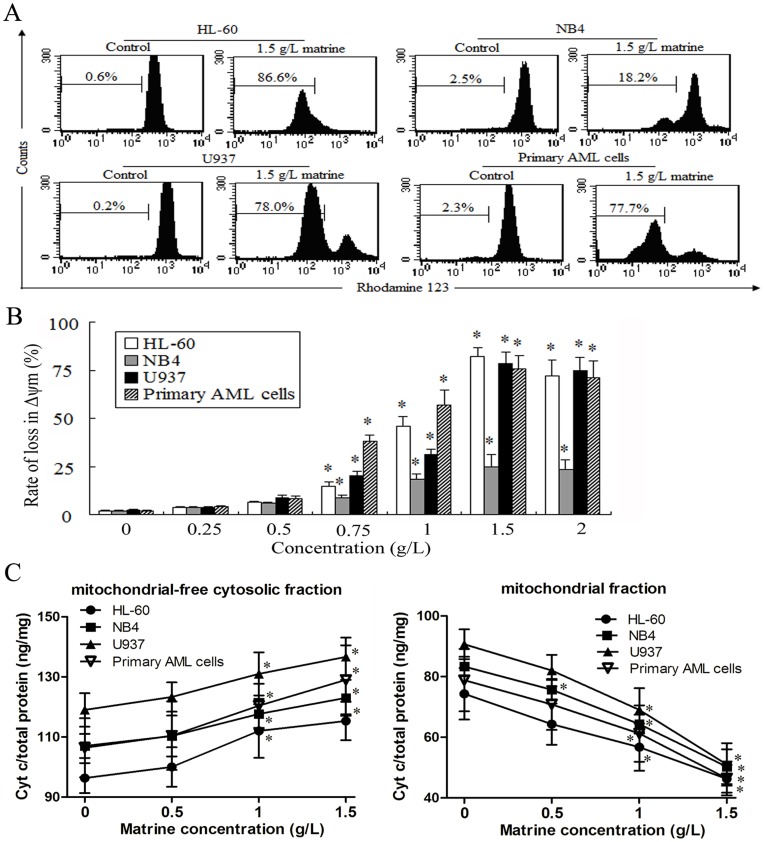
Effects of matrine on the changes of *Δψm* and cyt c in AML cells for 24 h. A. *Δψm* was analyzed by FCM using Rhodamine 123 dye, and representatives were shown in AML cells treated by 1.5 g/L matrine for 24 h. **B.** AML cells were treated with various concentrations of matrine for 24 h. **C.** The levels of cyt c in mitochondrial-free cytosolic fraction and mitochondrial fraction were detected by ELISA in AML cells. The values represent the mean ± SD of at least three independent experiments.^ *^ *P*<0.05 vs the respective control.

**Figure 4:**
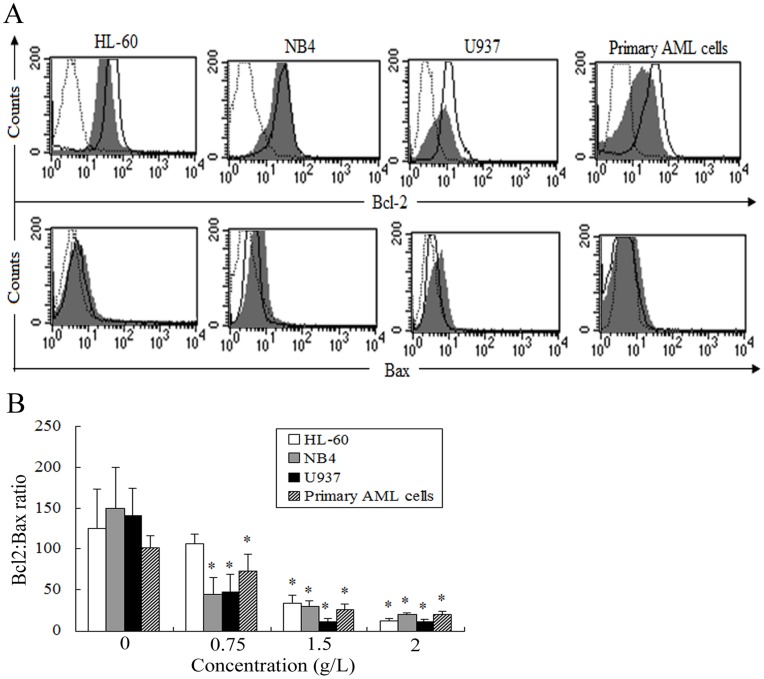
Effects of matrine on Bcl-2/Bax ratio in AML cells for 24 h. A. The expression of Bcl-2 and Bax were detected by FCM, and representatives were shown in AML cells treated by 1.5 g/L matrine for 24 h. **B.** AML cells were treated by various concentrations of matrine for 24 h. The values represent the mean ± SD of at least three independent experiments.^ *^ *P*<0.05 vs the respective control.

### Apoptosis Assay

AML cells were plated at a density of 2.0×10^5^ cells/well in 12-well plates. The cells were treated with various concentrations of matrine for 24 h or 1.5 g/L matrine for three different time points (12 h, 24 h, and 48 h). In addition, leukemic CD34^+^lin^−^ cells and normal CD34^+^lin^−^ cells were plated and treated with 1.5 g/L matrine for 24 h. Apoptosis was measured using FITC Annexin V Apoptosis Detection Kit II (BD Pharmingen™, San Diego, CA, USA) according to the manufacturer’s protocols. Data acquisition and analysis were performed using CellQuest software on a flow cytometry (FCM; FACSCalibur; BD, Mountain View, CA, USA). In addition, AML cells were treated with or without Z-Asp(O-Me)-Glu(O-Me)-Val-Asp(O-Me) fluoromethyl ketone (Z-DEVD-FMK; Sigma-Aldrich), a caspase-3 inhibitor for 1 h before treatment with matrine for 24 h, then, the apoptosis analysis was performed as described above.

**Figure 5:**
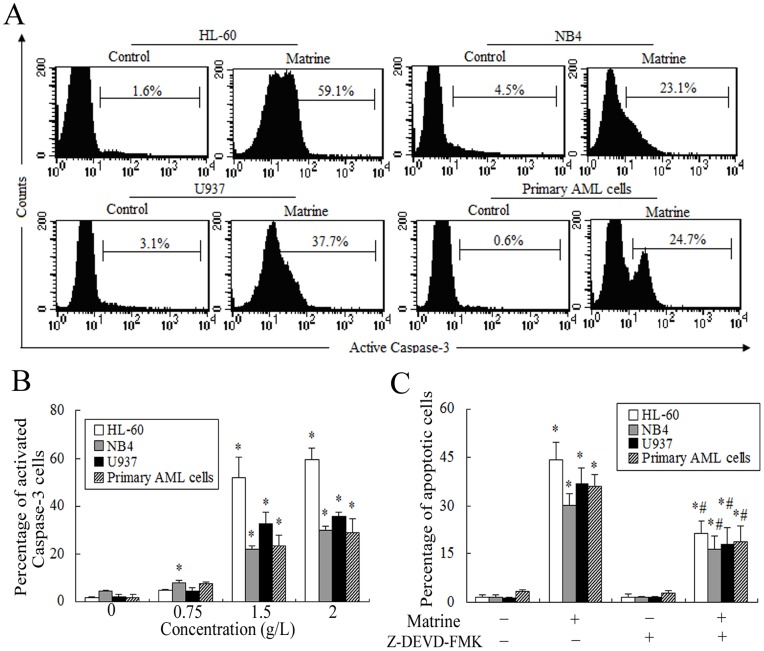
Effects of matrine on the activated caspase-3 in AML cells for 24 h. A. The expression of activated caspase-3 was analyzed by FCM and representatives were shown in AML cells treated by 1.5 g/L martrine for 24 h. **B.** AML cells were treated by various concentrations of matrine. **C.** Effects of Z-DEVD-FMK on matrine-induced apoptosis detected by annexin V-FITC binding and PI staining method. Apoptotic cells were determined after treatment with matrine (1.5 g/L) in the presence (+) or absence (−) of Z-DEVD-FMK (5 µM) for 24 h. The values represent the mean ± SD of at least three independent experiments.^ *^ *P*<0.05 vs the respective control, ^#^ *P*<0.05 vs the matrine only group.

### *Δψm* and Cyt c Assay

Rhodamine 123 is a yellow-green fluorescent probe that stains mitochondria in living cells in a membrane potential-dependent fashion. Cells were plated into 12-well plates and treated with matrine for 24 h. Then, cells were collected, washed twice with cold phosphate-buffered saline (PBS), and incubated with 5 µM Rhodamine 123 (Sigma-Aldrich) at 37°C in the dark for 30 min before FCM analysis. The cells were subsequently washed twice and analyzed by FCM. As described previously [21], [23], we measured the expression of Cyt c protein in mitochondrial-free cytosolic fraction and mitochondrial fraction with cyt c ELISA kit (Calbiochem, San Diego, CA, USA) according to the manufacturer’s protocols.

**Figure 6:**
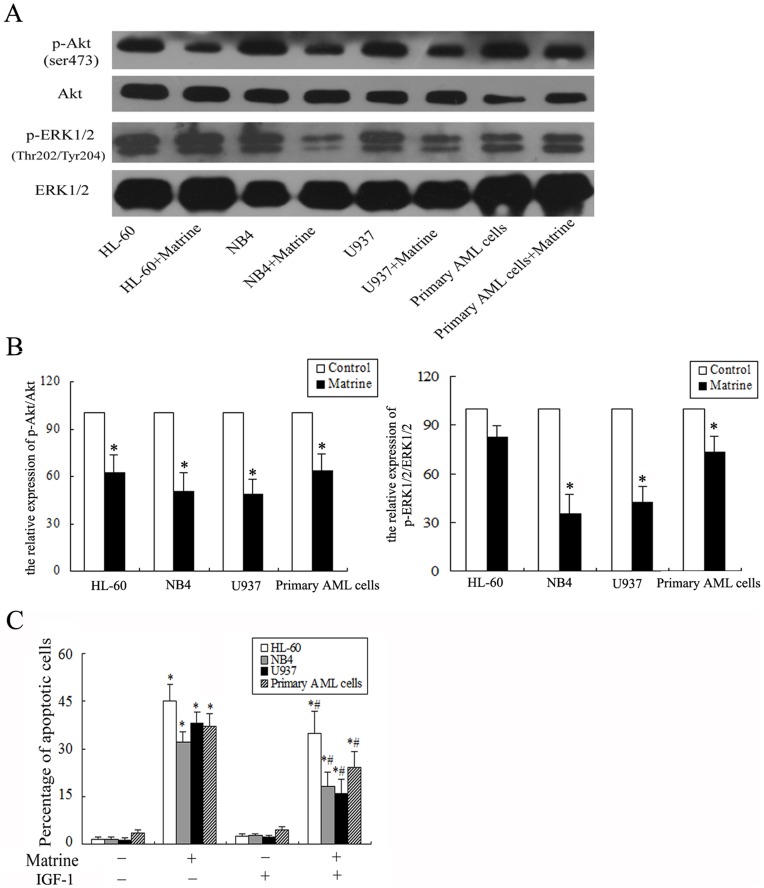
Matrine induced apoptosis through Akt and ERK1/2 inactivation in AML cells. A. AML cell lines (HL-60, NB4, and U937) and primary AML cells were exposed to 1.5 g/L matrine for 24 h, after which cell lysates were extracted and subjected to western blot analysis to monitor expression of Akt, p-Akt, ERK1/2, and p-ERK1/2. Representatives were shown. **B.** The optical densities of the bands were measured using Scion image analysis and data were presented as the mean ± SD of at least three independent experiments. **C.** Effects of IGF-1 on matrine-induced apoptosis detected by annexin V-FITC binding and PI staining method. Apoptotic cells were determined after treatment with matrine (1.5 g/L) in the presence (+) or absence (−) of IGF-1 (100 ng/ml ) for 24 h. The values represent the mean ± SD of at least three independent experiments.^ *^ *P*<0.05 vs the respective control, ^#^ *P*<0.05 vs the matrine only group.

### Bcl-2, Bax, and Activated Caspase-3 Assays

AML cells were plated into 12-well plates and treated with various concentrations (0, 0.75, 1.5 and 2 g/L) of matrine for 24 h. Then, the cells were collected, fixed, permeabilized, stained with Bcl-2 PE, Bax PE (Santa Cruz Biotechonoly, Santa Cruz, CA, USA), and activated caspase-3 PE (BD Pharmingen™), respectively. The expression levels were subsequently detected and analyzed by FCM.

**Figure 7:**
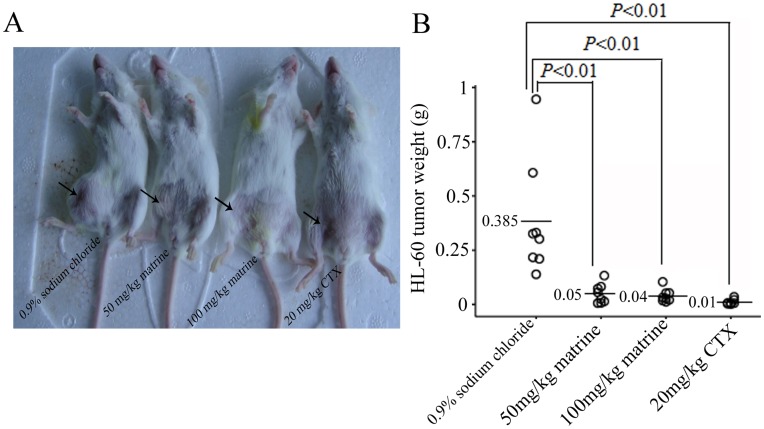
Matrine inhibit the growth of HL-60 cells *in vivo*. A. SCID mice with established HL-60 tumors were treated with either matrine (50 and 100 mg/kg, i.p.) or control (negative, 0.9% sodium chloride; positive, 20 mg/kg CTX). Matrine significantly reduced the tumor weight *in vivo*. One representative of eight mice in the each group was showed. **B.** The tumor weight in the each group. Each open circle represents a single individual assessed in the respective group and numbers on the left of horizontal bars represent the group means.

### Western Blot Analyses

After treatment with matrine, the related changes of protein expression in cell lysates were detected using western blot. Briefly, collected cells were lysed immediately in M-PER® Mammalian protein extraction reagent (Pierce, Rockford, IL, USA) supplemented with Halt protease and phosphatase inhibitor cocktail (Pierce). Protein concentration was assessed using BCA protein assay kit (Beyotime, Haimen, Jiangsu, China). Then, equal amounts of protein (30 µg) were boiled for 5 min, separated by SDS-PAGE, and electroblotted onto a PVDF membrane. After blocking, membranes were incubated with respective antibodies including Akt, p-Akt (Ser473), ERK1/2, and p-ERK1/2 (Thr202/Tyr204) (Cell signaling technology, Beverly, MA, USA) at appropriate dilutions overnight at 4°C. Membranes were then washed and incubated with HRP-conjugated secondary antibody (Santa Cruz Biotechnology) for 2 h at room temperature. Membranes were then washed again and developed using enhanced chemiluminescence. The optical densities of the bands were measured using Scion image analysis (Scion Cooperation, Frederick, MD, USA).

### *In vivo* Efficacy of Matrine

The severe combined immunodeficiency (SCID) mice (6–8 weeks old, 18–20 g body weight), bred in-house, were maintained throughout in specific pathogen-free (SPF) environment. Exponentially growing HL-60 (8×10^6^) were suspended in 100 µl PBS and subcutaneously injected into the right hind leg of recipient mice. On day 5, these mice were randomly divided into 4 groups, with 8 animals in each group. Then, the two treatment groups were injected intraperitoneally at two doses of matrine (50 mg/kg and 100 mg/kg) on alternative days, respectively. Positive and negative control group animals were given CTX (20 mg/kg) and 0.9% sodium chloride, respectively. All mice were killed on day 22 after these drugs had been administered seven times and tumor masses were carefully dissected out and weighted. Animal procedures were carried out in accordance with institutional guidelines after Wenzhou Medical College and Fudan University Animal Care and Use Committee approved the study protocol.

### Statistical Analysis

Data were presented as mean ± SD and were representative of at least three independent experiments. A one-way analysis of variance (ANOVA) was used to examine differences among the matrine groups with different doses. Differences were considered significant when *P*<0.05.

## Results

### Matrine Inhibited Proliferation of AML Cells

We examined the effects of matrine on the proliferation of AML cell lines HL-60, NB4, U937, and primary AML cells (BMMNCs). The positive expression rate of cytoplasmic myeloperoxidase was (82.1±6.2) % in BMMNCs from 9 patients with AML. Matrine inhibited the proliferation of AML cell lines HL-60, NB4, and U937 in a dose- and time-dependent manner, with IC_50_ at 24 h of 0.91, 0.66, and 0.59 g/L, respectively (Fig.1A, 1B, and 1C). Furthermore, matrine also inhibited the proliferation of BMMNCs, with IC_50_ at 24 h of 0.56 to 0.99 g/L (Fig.1D). 1.5 and 2 g/L matrine exhibited inhibitory effects on the proliferation of leukemic CD34^+^lin^−^ cells (Fig.1E). However, only 2 g/L matrine was found to have a slight inhibitory effect on the proliferation of normal CD34^+^lin^−^ cells (Fig.1F). The results indicated that matrine could potently inhibit the proliferation of AML cell lines as well as primary AML cells.

### Matrine Induced Apoptosis in AML Cells

The ability of matrine to induce AML cell apoptosis was assessed by Annexin V-FITC/PI double staining. After treatment with various concentrations of matrine for 24 h, the maximal apoptosis rates were 41.1% in HL-60 cells, 33.0% in NB4 cells, 37.2% in U937 cells, and 33.4% in primary AML cells, respectively (Fig.2A and 2B). In contrast, less than 5% of untreated AML cells underwent apoptosis under the same conditions. Obviously, the optimal concentration of matrine induced apoptosis in AML cells was 1.5 g/L. Furthermore, we also detected the changes of apoptosis with different treatment times. The results demonstrated that the apoptosis rates of AML cells were approximately 10% at 12 h and 37% at 24 h after treatment with 1.5 g/L matrine, then, the percentage of apoptotic cells decreased (Fig.2C). The apoptosis rate of leukemic CD34^+^lin^−^ cells treated with 1.5 g/L matrine for 24 h was 5.1% (Fig.2A), which suggested that matrine can slightly induce apoptosis in leukemic CD34^+^lin^−^ cells. However, no significant apoptosis occurred in normal CD34^+^lin^−^ cells treated with 1.5 g/L matrine for 24 h compared to the controls (Fig.2A), which suggested that matrine has no effect on the induction of apoptosis in normal CD34^+^lin^−^ cells. The results indicated that matrine could induce apoptosis in AML cell lines as well as primary AML cells.

### Matrine Disturbed *Δψm* and Induced Cyt c Release from Mitochondria to Cytosol in AML Cells

Mitochondria play a key role in the regulation of apoptosis, and changes in *Δψm* reflect mitochondrial dysfunction. In this study, we measured *Δψm* using Rhodamine 123 after treatment with matrine. As shown in Fig.3A and Fig.3B, matrine induced loss of *Δψm* in a dose-dependent manner, and more than 75% of HL-60 cells and U937 cells as well as primary AML cells decreased in *Δψm* after treatment with 1.5 g/L matrine for 24 h. However, after treatment with matrine for 24 h, only 25% of NB4 cells lost in *Δψm*. Consisted with the results of *Δψm*, the levels of mitochondrial cyt c were significantly decreased and the amounts of mitochondrial-free cytosolic cyt c were markedly increased (Fig.3C). These results indicated that apoptosis might be induced by matrine via the mitochondrial pathway in AML cells.

### Matrine Reduced the Ratios of Bcl-2/Bax Protein in AML Cells

AML cell lines and primary AML cells express high levels of Bcl-2 and low levels of Bax (Fig. 4A). After treatment with various concentrations of matrine (0.75, 1.5, and 2.0 g/L) for 24 h, the expression levels of Bcl-2 decreased while the expression levels of Bax increased in AML cell lines as well as primary AML cells. Thus, the Bcl-2/Bax ratio was markedly down-regulated (Fig. 4B). After treatment with 1.5 g/L matrine for 24 h, the expression of Bcl-2 protein decreased about 30% in U937 cells, while a small decrease occurred in HL-60, NB4 and primary AML cells, with a concomitant significant increase in the expression levels of Bax in AML cells. These results indicated that apoptosis might be induced by matrine via regulating the Bcl-2/Bax ratio in AML cells.

### Matrine Induced Apoptosis of AML Cells via Activation of Caspase-3

We investigated the effect of matrine on the expression of activated caspase-3, in order to research whether matrine-induced apoptosis of AML cells was dependent on activation of caspase-3. The expression levels of activated caspase-3 significantly increased in AML cell lines (HL-60, NB4, and U937) and primary AML cells after treatment with high concentrations of matrine (1.5 and 2.0 g/L) for 24 h (Fig. 5A and Fig. 5B). In addition, it was examined whether apoptosis induced by matrine was affected by Z-DEVD-FMK, a caspase-3 inhibitor. Matrine-induced apoptosis in AML cells were partially diminished by the addition of Z-DEVD-FMK (Fig. 5C), which further confirmed the participation of caspase-3.

### Matrine Induced Apoptosis in AML Cells through Akt and ERK1/2 Inactivation

To investigate whether the Akt pathway and the ERK pathway involved in matrine-induced apoptosis, AML cells were treated with 1.5 g/L matrine for 24 h. Then, the expression levels of p-Akt and p-ERK1/2 were analyzed using western blot. As shown in Fig. 6A and 6B, matrine down-regulated the expression levels of p-Akt and p-ERK1/2 in HL-60 cells, NB4 cells, U937 cells, and primary AML cells. Furthermore, it was confirmed by IGF1, a potent activator of PI3K/Akt. 100 ng/ml IGF-1 partially decreased the apoptosis in AML cells (Fig. 6C), suggesting the Akt pathway involved in matrine-induced apoptosis.

### Effect of Matrine on Tumor Growth *in vivo*

We further studied the effect of matrine-mediated AML cell growth inhibition *in vivo*. The heterogeneous AML model was established in SCID mice following subcutaneous transplantation of HL-60 cells. As shown in Fig. 7A and 7B, administration of 50 mg/kg and 100 mg/kg matrine resulted in a significant regression of tumor weight, and the inhibitory rates were 87.0% and 89.6%, respectively. The inhibition levels of tumor growth between 50 mg/kg and 100 mg/kg matrine-treated groups have no statistically significance (*P*>0.05). The 20 mg/kg CTX-treated mice had the smallest tumor weight among 4 groups. These results indicated that matrine has a significant anti-leukemic effect *in vivo*.

## Discussion

The present study has shown that matrine induces marked growth inhibition and apoptosis in AML cell lines as well as primary AML cells *in vitro* and subcutaneous xenograft HL-60 cells in mice. Furthermore, we also exhibited a mechanism of action by which matrine induced apoptosis of AML cells via the mitochondrial-mediated pathway. In addition, it has been demonstrated that only a slight effect was observed on normal CD34^+^lin^−^ cells and peripheral blood mononuclear cells treated by matrine less than 2 g/L in our present and previous study [21], respectively. Thus, to our knowledge, this is the first time to report the effect of matrine on AML cells *in vitro* and *in vivo*.

Uncontrolled proliferation is a key aspect of tumorigenesis, and inhibiting cell proliferation can achieve growth arrest in tumor cells. The proliferation of AML cell lines HL-60, NB4, and U937 was significantly inhibited by matrine. We also showed that matrine had a marked cytotoxicity in BMMNCs from 9 patients with AML, in which included approximately 80% primary AML cells. We and others have reported that apoptosis is a key mechanism by which matrine kills tumor cells [20], [21], [24]. In our present study, the rates of apoptosis were more than 30% in AML cells tested after treatment with 1.5 g/L matrine for 24 h. Necrosis would happen as well as apoptosis in AML cells treated by matrine at too high a concentration or for too long a time. These results suggested that matrine inhibited cell proliferation of AML cells via the apoptosis pathway.

It has been widely recognized that apoptosis is initiated by two principal pathways: the mitochondria-mediated intrinsic pathway and the death-receptor-induced extrinsic pathway [25]. Mitochondria play a central role in apoptosis. During mitochondria dysfunction, the mitochondrial permeability transition pore (MPTP) open and cyt c release from mitochondria to cytosol. The opening of MPTP is controlled by the Bcl-2 family members. The Bcl-2 family members can be classified into three subfamilies, anti-apoptotic members such as Bcl-2, Bcl-XL, and MCL1, pro-apoptotic members such as Bax and Bak, and the BH3-only members such as Bim and Bad [26]. Bcl-2 is present in the outer mitochondrial membrane, where function to suppress apoptosis via blocking cyt c release and binding to Apaf-1. In the presence of excess Bax, Bcl-2 is displaced from Apaf-1, which may promote apoptosis. In our present study, a decrease of the ratios of Bcl-2/Bax protein occurred in AML cell lines HL-60, NB4, U937 as well as primary AML cells after treatment with matrine, which caused *Δψm* loss and cyt c leak out.

The release of cyt c from the mitochondrion plays a crucial role for the execution of the apoptotic pathway because it activates caspase-9, which subsequently activates caspase-3. The activated caspase-3 leads to the final destruction of the target cell. We have shown that the levels of activated caspase-3 of AML cells induced by matrine increased in a dose-dependent manner. The activation of caspase-3 occurred concomitantly with apoptosis in AML cells, suggesting that the activation of caspase-3 might lead to apoptosis. This viewpoint was further verified by the addition of Z-DEVD-FMK. Combined with with 1.5 g/L matrine and 5 µM Z-DEVD-FMK, apoptosis was partially blocked. These results indicated that matrine-induced apoptosis in AML cells was partially dependent on the activation of caspase-3.

Some signaling pathways, such as PI3K/Akt and ERK/MAPK, play a critical role in the pathogenesis and progression of AML [27], [28]. The deregulation of these signaling pathways promotes the survival and proliferation of hematopoietic progenitor cells. Furthermore, it has been reported that AML patients with the overactivation of Akt signaling in AML cells have a worse prognosis and shorter survival compared to those patients with normal levels of Akt activation [29]. Inactivation of Akt signaling has been shown to inhibit proliferation and induce apoptosis in many other cell types [30]–[32]. Similarly the present data demonstrated that matrine suppressed the phosphorylation of Akt in all AML cells tested. Combined the result treated with matrine and IGF-1 in AML cells, it was further verified that matrine-induced apoptosis can be attributed, at least partially to Akt inactivation. The discovery of matrine as a novel Akt inhibitor may have implications for cancer biology and treatment. In addition, we have demonstrated that matrine inactivates the phosphorylation of ERK1/2 in NB4, U937, and primary AML cells during matrine-induced apoptosis, which differ from the previous report [19]. Liu *et al* [19] showed that the expression levels of p-ERK1/2 had no change at 48 h after treatment with 0.4 g/L matrine in U937 cells. Differences may be explained by the different concentration matrine chosen.

In conclusion, matrine exerts a significant anti-leukemic effect on AML cells *in vitro* and *in vivo*. The anticancer activity of matrine could be attributed to its inhibition of proliferation and apoptosis induction of AML cells through mitochondria-mediated pathway and inhibition of the Akt and ERK1/2 pathways. Given our findings that matrine inhibits proliferation and induces apoptosis in AML cell lines and primary AML cells, matrine may be a useful candidate as chemotherapeutic agent against AML.
